# Accurately decoding visual information from fMRI data obtained in a realistic virtual environment

**DOI:** 10.3389/fnhum.2015.00327

**Published:** 2015-06-09

**Authors:** Andrew Floren, Bruce Naylor, Risto Miikkulainen, David Ress

**Affiliations:** ^1^Electrical and Computer Engineering Department, The University of Texas at AustinAustin, TX, USA; ^2^Department of Neuroscience, The University of Texas at AustinAustin, TX, USA; ^3^Department of Computer Science, The University of Texas at AustinAustin, TX, USA; ^4^Human Neuroimaging Laboratory, Baylor College of MedicineHouston, TX, USA

**Keywords:** fMRI BOLD, machine learning, human vision, virtual environments, natural stimuli

## Abstract

Three-dimensional interactive virtual environments (VEs) are a powerful tool for brain-imaging based cognitive neuroscience that are presently under-utilized. This paper presents machine-learning based methods for identifying brain states induced by realistic VEs with improved accuracy as well as the capability for mapping their spatial topography on the neocortex. VEs provide the ability to study the brain under conditions closer to the environment in which humans evolved, and thus to probe deeper into the complexities of human cognition. As a test case, we designed a stimulus to reflect a military combat situation in the Middle East, motivated by the potential of using real-time functional magnetic resonance imaging (fMRI) in the treatment of post-traumatic stress disorder. Each subject experienced moving through the virtual town where they encountered 1–6 animated combatants at different locations, while fMRI data was collected. To analyze the data from what is, compared to most studies, more complex and less controlled stimuli, we employed statistical machine learning in the form of Multi-Voxel Pattern Analysis (MVPA) with special attention given to artificial Neural Networks (NN). Extensions to NN that exploit the block structure of the stimulus were developed to improve the accuracy of the classification, achieving performances from 58 to 93% (chance was 16.7%) with six subjects. This demonstrates that MVPA can decode a complex cognitive state, viewing a number of characters, in a dynamic virtual environment. To better understand the source of this information in the brain, a novel form of sensitivity analysis was developed to use NN to quantify the degree to which each voxel contributed to classification. Compared with maps produced by general linear models and the searchlight approach, these sensitivity maps revealed a more diverse pattern of information relevant to the classification of cognitive state.

## Introduction

Recent research has shown that fMRI is capable of decoding some cognitive states (Mitchell et al., 2004) such as the cognitive states associated with the perception of various types of objects (Shinkareva et al., 2008; Cabral et al., 2012), what a person is saying and who is saying it (Formisano et al., 2008a), and telling the truth or lying (Fan et al., 2006). The ability to decode cognitive states during training and therapy exercises could be invaluable for improving their efficacy. Virtual environments (VEs) are the most practical way to perform such exercises within the confines of an MRI scanner, and a number of virtual training and therapy environments already exist (Gerardi et al., 2008; Gonçalves et al., 2012). However, these exercises are far from the controlled stimuli used in most fMRI experiments. During such natural tasks, we expect a variety of complex interactions between many regions of the brain. A goal of the work reported in this paper is further development of computational analysis techniques that improve decoding accuracy of cognitive states in such an environment. Rather than focusing on a specific set of cognitive states, we look to develop a general approach to decoding task-relevant states with high accuracy. Additionally, the visual richness, the motion of the objects and viewer, and the real-time interaction with the virtual environment bring experiences to the subject much closer to those which shaped the evolution of our brains. It seems plausible then that using VEs can reveal how the brain functions under more realistic circumstances. Therefore, another goal of the work reported in this paper is to further development of analysis techniques that improve the interpretability of complex decoding algorithms for use in hypothesis driven experiments.

Using VEs in fMRI experiments have been explored by various researchers over the past decade. Early examples can be found in the work of Spiers and Maguire (2007b) and the resulting publications (Valente et al., 2011) from the PBAIC 2007 competition (see http://www.lrdc.pitt.edu/ebc/2007/competition.html). In the case of Spiers and Maguire, a commercial taxi driving game was used as the stimulus, thereby leveraging many millions of dollars in development expense, but at the same time severely limiting control of the stimulus by the researchers. The game play was recorded during scanning, and afterwards the subject reviewed the video with a researcher and explained what they were thinking and doing at each point to assist in labeling the data. For the PBAIC 2007 competition, researchers constructed an interactive VE using the Source game engine. Subjects were given a relatively complex task to search for fruits, toy weapons, and characters with piercings, while avoiding contact with a dog. Subjects received compensation after the scan based on the score they received in the game. Similar to the work of Spiers and Maguire, the game play was recorded and participants rated their subjective mood along several axes, including arousal and valence, while reviewing the video. More recently, researchers have begun using VEs in more traditional controlled experimental protocols utilizing specially designed and far simpler VEs (Marsh et al., 2010; Mueller et al., 2012; Op de Beeck et al., 2013; Schindler and Bartels, 2013). These stimuli cost much less than a commercial game, and so are commensurately less realistic.

For our experiments, we used a virtual environment specially developed for us by a professional game and simulation designer using a state-of-the-art game engine. The visual quality of the environment and the motion of characters and camera were comparable to what is found in military training systems. The visual quality is similar to the stimuli used in the work of Spiers and Maguire as well as the PBAIC 2007 competition. However, in those stimuli the induced cognitive states are not well balanced. Due to their interactive nature, the subjects may spend significantly more time in one state than another. This complicates the training and, in particular, the evaluation of decoding algorithms. In our stimulus, we have balanced the induced states at the cost of interactivity to provide better accuracy estimates of different decoding methods for comparison. On the other hand, the stimulus is considerably more realistic—and the subject's state less controlled—than what is found in the recent neuroscientific investigations involving VEs (Marsh et al., 2010; Mueller et al., 2012; Op de Beeck et al., 2013; Schindler and Bartels, 2013). It was important to measure the performance of different decoding methods in this environment to gauge their potential for use with training and therapy exercises.

**Figure 1:**
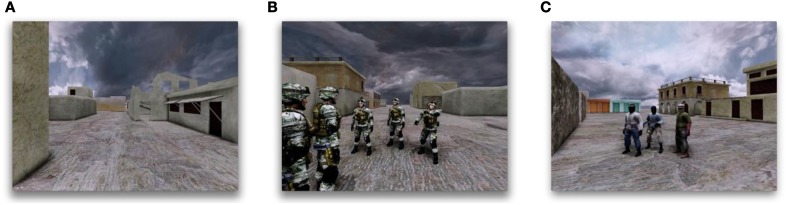
**The stimulus in the experiment described in this paper employs a virtual environment and a blocked design where the view alternates between moving through the environment and viewing groups of animated characters. (A)** An example frame from the stimulus where the camera is traveling through the virtual environment with no characters presented. **(B)** An example frame from the stimulus where five friendly characters are being presented. **(C)** An example frame from the stimulus where three hostile characters are being presented. Such stimuli allow studying how the brain responds in a more natural and complex environment.

Our goals were focused on exploring and improving methods of data analysis coupled with virtual environment stimulus design, rather than testing a specific neuroscience hypothesis. We aimed to extract the cognitive state of the subject associated with freely viewing a number of characters, rather than test the many possible perceptual mechanisms that encode this information in the human brain, such as object recognition, eye movements, or social group perception. Such decoding methods will be important for use of fMRI in clinical settings where it is useful to know the task-relevant cognitive state of the subject, but the neural mechanisms may not be well understood yet. We are, for example, interested in supporting work using virtual reality to treat PTSD due to combat, in which treatment exposes the subject to virtual stimuli that are highly suggestive of the physical situations that induced the trauma. Through carefully controlled use of VR, the patient is gradually desensitized over a period of weeks so that the likelihood of triggering of the trauma declines (Gerardi et al., 2008), as confirmed through fMRI measurements (Gonçalves et al., 2012). Our stimulus and experiments were developed with this in mind. In particular, we created a virtual town suggestive of the Middle East, and populated the town with a combination of U.S soldiers and foreign combatants (Figure 1).

Most neuroscience experiments analyze their data using hypothesis-based statistical techniques, such as the general linear model (GLM), Such methods can be very effective only when a distinct and testable hypothesis is available. However, the closer the stimuli get to realistic experiences, as offered using VE, the more difficult it becomes to isolate a tractable hypothesis. Moreover, it is likely that the more complex VE stimuli will evoke a more broadly distributed cortical response that includes both low-level sensory and higher-level associative regions. The treatment of each voxel independently by GLM cannot capture the structure of multi-voxel responses reflecting the coordinated activity these widely distributed brain regions. For all these reasons, we employ multi-voxel pattern analysis (MVPA) based on machine learning, an approach introduced in Haxby et al. (2001).

We offer a new combination of methods to decode and analyze VE stimulus information from fMRI data. Most fMRI applications of machine learning have shown discrimination between distinct object categories (Haxby et al., 2001; Pereira et al., 2009). More recently the relationship between multiple objects has been explored (Baeck et al., 2013). Here we demonstrate that the cognitive state associated with object number rather than object classification can be decoded from fMRI data. Specifically, the cognitive state associated with viewing a number of animated characters, varying from 1 to 6 can be decoded in a dynamically changing virtual environment with accuracy from 58 to 93% (chance is 16.7%). Such high classification accuracy has important potential for real-time fMRI based therapies that adjust the stimulus in response to brain activity.

To achieve this performance, we experimented with four machine learning algorithms. We were particularly interested in artificial neural networks (NN) and support vector machines (SVM). For completeness, we also tested a Gaussian naive Bayes classifier (GNB) (Duda and Hart, 1973), and k-nearest neighbor classifier (KNN). The SVM is the most commonly used machine-learning algorithm in MVPA analyses (Pereira et al., 2009). However, we found that NNs also produced very favorable results. Recent advances in NNs, such as deep learning (Hinton et al., 2006) and convolutional networks, have been outperforming traditional SVMs in a variety of domains (Cirşan et al., 2012). Before jumping to these advanced techniques, we wanted to explore the application of relatively simple feed-forward NNs on fMRI data, and we propose several methods for improving their classification performance.

MVPA classification performance can tell us to what degree the time-series data can be used to decode a target category, but we also want to know which voxels are encoding the desired stimulus information. The searchlight technique (Kriegeskorte et al., 2006) can be used in conjunction with any machine-learning algorithm to create a map, but it does not fully utilize the spatially distributed multivariate nature of the classifier. For SVMs, the absolute discriminative map (Formisano et al., 2008b) has been used. However, the discriminative map is limited to SVM algorithms. We propose a new mapping method similar to the absolute discriminative map based on a technique called sensitivity analysis (Zurada et al., 1994). For an SVM, the method reduces to approximately the absolute discriminative map. However, the method is more general and has been adapted to the NN. The sensitivity analysis is used in several ways. First, we examine the use of the sensitivity results to train NNs on high-dimensional fMRI data with relatively few training examples. Second, we present a method for producing informative maps from trained networks based on sensitivity analysis. Finally, we develop a technique based on recursive feature elimination (Guyon et al., 2002) to determine appropriate thresholds for these sensitivity maps. The recursive feature elimination technique also acts as a multivariate feature reduction technique that can improve decoding performance. A similar method was applied to SVMs in the work of De Martino et al. (2008).

## Methods

### Subjects

Five adult males, ages 24–57, with normal or corrected-to-normal vision, participated in the experiments. All subjects participated in two fMRI sessions and a third session to acquire a high-resolution structural anatomy. Informed consent was obtained from all subjects under a protocol approved by the University of Texas at Austin Institutional Review Board.

### Stimulus

For designing our virtual environment, we used the Unreal Developer's Kit developed by Epic Games, Inc.. This development kit is available free of charge for non-commercial applications (http://www.unrealengine.com/udk) and uses the same rendering and game engine found in many current and popular video games.

We created a virtual environment suggestive of a town in the Middle East (Figure 1). The stimulus was rendered in real-time from the point of view of a camera moving at eye level through the town, providing a first-person-perspective experience. Virtual characters representing friendly forces and hostile combatants were situated at four locations in the town. The camera would travel steadily on a predefined path from one part of the town to another over a 15 s interval during which no characters were visible. When one of the pre-determined locations was reached, characters would appear for 15 s during which the camera panned back-and-forth slowly while keeping all the characters in the field-of-view. The characters engaged in simple repetitive animated movement sequences. The number of characters presented at each of these locations varied from one to six, but the number and position of the characters did not vary over each 15-s period (Figures 1B,C). The 30-s block design of the stimulus was used for feature selection and for improving classifier performance (see below). The MVPA classification was always applied only to the periods of time when characters were presented. While the stimulus followed a 30-s block design, the subject always had the context of being present in the virtual town. This was done in order to preserve as much realism as possible by avoiding a discontinuity in context induced by using a blank screen for contrast.

A scanning session of a single subject entailed five to six “runs,” where each run was 6 min in duration. During a single run, every possible number of characters, from one to six, was presented twice, for a total of 12 presentations of characters (15 s each). The order of the number of characters was generated by a random permutation of 1–6 applied twice, once for the first half of the run and once for the second half. Since we placed characters at only four different locations in the town, the camera made three loops through the town in order to provide 12 presentations of characters. Finally, the type of character, soldiers or insurgents, was the same through a given run, and this character type was alternated between runs.

The subjects were not given any specific instructions other than to view the scene. The subjects were not military personnel, and they were not instructed to perceive specific characters as friendly or hostile. No background context was provided to bias the way the subjects perceived the environment.

Our VE stimulus was designed to reduce low-level visual differences between the group-number categories. First, the imagery was never static. Once again, achieving as much realism as possible motivated this decision. It is well known that the visual experience of looking at still-imagery is quite different from looking at moving imagery (Spiers and Maguire, 2007a). Second, the distance and viewpoint of the character groups was randomized from presentation-to-presentation to reduce low-level contrast differences between character-number categories. In addition, the subject performed free viewing of the scene, so exploratory eye movements should average away any local contrast correlations with character number. However, it is possible that the total contrast across the scene is correlated with character count, as this would not be affected by eye movements. Therefore, we calculated total scene contrast for each frame of the stimulus using the root-mean-square (RMS) contrast measure (Kukkonen et al., 1993). We then fit a GLM to the result with character counts 0–6 as explanatory variables and performed a *t*-test on each of these variables to determine if a significant correlation existed between total scene contrast and character count (Friston et al., 1994). To determine what effect any such correlations, whether significant or not, had on the performance of the classifiers, we trained an SVM on the total contrast data and compared the performance with the fMRI data. Performance was estimated using two-fold cross-validation. Since the total contrast is identical for each run there are only two possible folds that are unique and contain every character count.

### MRI protocols

Imaging was performed on a GE Signa Excite HD 3T scanner using the product eight-channel head coil. Whole-brain image volumes were collected using a custom GRAPPA EPI sequence (Griswold et al., 2002). Sequence parameters were *g*-factor = 2, TE = 25 ms, TR = 2.5 s, and 2.5-mm cubic voxels across a 200 mm field-of-view. The slice prescription included 40 slices oriented along the AC-PC axis. A high-order shim was performed before the start of the functional imaging to improve field homogeneity.

A set of T1-weighted structural images was obtained on the same prescription before the functional acquisition runs using a three-dimensional (3D) fast RF-spoiled gradient-echo (fSPGR) sequence. These anatomical images were then used to align the functional data to a structural 3D reference volume, which was acquired for each subject in a separate session. The structural reference volume was T1-weighted with good gray-white contrast and was acquired using a 3D inversion-prepared fSPGR sequence (minimum TE and TR, TI = 450 ms, 15° flip angle, isometric voxel size of 0.7 mm, 2 excitations, ~28-min duration).

### Preprocessing

Preprocessing of the fMRI data was performed using the mrVista software package (available at http://vistalab.stanford.edu/), modified for use in our own lab. The first 15 s of data were discarded to reduce transient effects. Within-scan motion was then estimated using a robust intensity-based scheme (Nestares and Heeger, 2000). Between-run motion was corrected using the same scheme, this time applied to the temporal average intensity of the entire scan. The first run of the session was used as the reference. Because the goal is to learn associations between patterns of activation in the brain and stimulus presentation, it is important that the activation is temporally aligned with the stimulus. Therefore, a Wiener filter deconvolution (Poor, 1980) was applied using a generic difference-of-gamma hemodynamic response function (Glover, 1999) as the kernel to the recorded BOLD signal. Mostly, the deconvolution served to shift the peak response in time so that it was aligned with its associated stimulus, but it also provided some amount of noise reduction. The high-resolution reference anatomies were segmented using the Freesurfer image analysis suite (http://surfer.nmr.mgh.harvard.edu/) to create approximate parcellations of the gray matter in each subject, as well as a surface model for visualization of mapping results.

### Cross-validation

The performance of machine learning algorithms is generally defined to be the expected accuracy of the classifier on previously unseen examples (Bishop, 2006). In practice, this measure can only be estimated. A typical approach is to split the available examples into training and test sets. The classifier is first trained on the training set, and its performance on the test set is then taken as the estimate of classifier performance on future unseen data. The splitting process is performed multiple times to reduce the variance of the performance estimate. This procedure is known as k-fold cross-validation (Kohavi, 1995).

In order to generate *p*-values for these performance estimates, the null distribution for the cross-validated performance was generated by randomly permuting the labels on the examples 2000 times and repeating the training and cross-validation procedure. That is, the distribution of performance estimates was generated under the assumption that the labels and data were independent. Using this distribution, *p*-values were calculated for the performance estimates (Ojala and Garriga, 2010). The high-performance computing resources of the Texas Advanced Computing Center at The University of Texas at Austin were utilized to perform this computation.

Previous studies (Pereira et al., 2009) have raised issues with performance estimates that are optimistically biased due to temporal correlations between examples (time frames) that violate standard assumptions of independence between training and test sets. For fMRI, the hemodynamic response introduces temporal correlations on the order of 10 s, which raises the question: What is the relationship between performance estimates and temporal correlation? To address this question, we estimated classifier performance when classifying number of characters presented using four different methods for splitting the data between training and test sets. Frames where no characters were present were removed, leaving 72 frames per run. We grouped different numbers of the remaining consecutive frames into selection units: 1 frame (*frame split*), 6 frames (*block split*), 36 frames (*half-run split*), and 72 frames (*run split*). For each of these unit sizes, we formed training and test sets by randomly selecting individual units (without replacement) and estimated classifier performance using these sets. For the frame and block splits, classifier performance was estimated using ten-fold cross-validation. For the half-run and run splits, only eight- and four-fold cross-validation was used respectively, due to the limited number of runs per subject. Based on our results, we chose to utilize the block split for performance estimates, as it did not exhibit an optimistic bias and allowed us to use more folds in the cross-validation procedure, which reduces the variance of the performance estimates.

### Feature selection

We used feature selection methods to remove uninformative voxels, thus improving both training time and performance of the machine-learning algorithms. This was particularly important for the NN, where training times can be quite long compared to the other methods. Common tools for feature selection in neuroimaging include anatomical region-of-interest (ROI) selection, principal component analysis (PCA; Hotelling, 1933), and univariate statistical tests. ROI selection is a powerful aide for hypothesis testing, but is much less useful for data exploration. PCA selects the orthogonal projections with the highest variance, which are generally dominated by physiological nuisance and is therefore not well suited for our purposes. Instead, we used ANOVA (Scheffe, 1959), which has been shown to be effective for feature selection in the context of MVPA (Norman et al., 2006; Pereira et al., 2009). The idea behind ANOVA is to calculate the mean and variance for the set of samples in each class (e.g., number of characters), and then use these statistics to determine how different the distributions for each class are. We used ANOVA in one of two different ways: selecting voxels that differed significantly between with-character and without-character periods, or selecting voxels that differed significantly across classification targets (i.e., number of characters). To calculate significance, ANOVA estimates the probability that the means of two different samples are different. For comparison, we performed both task-activated feature selection and classification-target feature selection. We found classification-target feature selection yielded the best results on this dataset. Additionally, care must be taken to avoid optimistically biasing the accuracy estimates; voxel selection must be performed within each fold of a cross-validation procedure.

### Classification

Using the time series from the voxels selected by the ANOVA process, we constructed classifiers of the following types: one-vs.-one multi-class linear support-vector machine with *C* = 1 (Cortes and Vapnik, 1995; Weston and Watkins, 1999), feed-forward neural network with scaled conjugate gradient backpropagation training (Hornik et al., 1989; Møller, 1993; Hagan and Menhaj, 1994), Gaussian naive Bayes classifier (GNB) (Duda and Hart, 1973), and *k*-nearest neighbor classifier (KNN) with *k* = 6 (Cover and Hart, 1967). The parameters for the SVM and KNN were determined by a grid-search (Hsu et al., 2010) on a left-out dataset. That is, the parameters were obtained on data not used in the cross-validation procedure to estimate performance. The performance of each classifier was estimated for three different classification problems: whether characters were present, how many characters were present, and what type of characters was present. For the former classification, the full time series was utilized; for the latter two classifications, we used only the fMRI data obtained during the character-present periods. The number of examples for each label was always balanced.

Although the NN can potentially learn more complex classification functions than the other algorithms, it uses a stochastic training process and has many more free parameters. To overcome these issues, we performed model selection within each fold of the cross-validation procedure using a validation phase. First, part of the training data was held out as the validation set. We then performed a grid-search on the number of hidden nodes and selected the best value based on the network's performance on the held-out validation set. Then, using this hidden-node value, we trained 20 more networks and again selected the best network based on performance on the held out validation set. This procedure reduced considerably the variance of the NNs cross-validated performance.

### Classifier probability and confidence

All classifiers return a label for an input, but not all classifiers return the probability that the label is correct. For example, the SVM can only return a label, whereas the GNB classifier and feed-forward NN can return the probability for all labels (Richard and Lippmann, 1991). Normally, one chooses the label with the maximum probability as the selected class while ignoring its value, but we explored the use of this probability information to improve classification accuracy. It is also useful to consider a heuristic, which we shall call *confidence*, which is correlated with the probability that the chosen label is correct. For the GNB classifier, the probability of the chosen label can be used directly. However, the output of the NN is only an approximation of the posterior probabilities. Therefore, the outputs are first normalized to sum to one across all labels, and then the output corresponding to the selected label is taken to be that label's confidence. Since the SVM only returns a label, generating a measure of confidence is not as straightforward and we therefore elected to only measure the confidence of the NN. How well confidence correlates with the true probability depends on how well the NN has approximated the joint probability distribution after training. The true probability cannot be measured directly, but we can compare the average confidence with the average probability that a label is correct, that is, the estimated classifier accuracy. To see how well the NN is estimating the joint probability distribution, we averaged confidence across all frames in a session and plotted it against the session's cross-validated performance. It is also worth noting that confidence is calculated from the output of a trained NN and an input example, but not the associated label. This means that confidence could potentially be used as an independent quality estimate if the neural network was trained on an independent dataset.

### Block integration

A common approach to boost classification accuracy is to average across frames in a stimulus block (e.g., Pereira et al., 2009). We compared the use of individual frames as examples to the use of examples created by averaging across 15-s blocks, and found that the block-averaged examples produced better classifier performance. Block averaging exploits our prior knowledge about the temporal structure of the stimulus, but it is not the only alternative.

We explored three other approaches for exploiting this knowledge: block voting, confidence voting, and output averaging. Block voting can be applied to any machine-learning algorithm. In block voting, the classifier was trained using individual frames as input examples, but the classification of a block was chosen as the majority classification of all frames in that block – each frame in the block “votes” on the block classification. The block voting procedure can be interpreted as a median filter on the output of a classifier trained on individual frames, *c_block_* = Median[*c*_1_ *c*_2_ … *c*_6_] where *c_block_* is the classification of the block and *c*_1_, *c*_2_, …, *c*_6_ are the classifications of the individual frames in the block. Confidence voting requires an algorithm that returns a probability along with the label. Confidence voting was similar to block voting, but each frame's vote was weighted by the probability of the chosen label on that frame, $c_{block}=\arg\;\max_{c\epsilon \Omega }\sum_{i=1}^{6}w_{i}\cdot 1_{c}(c_{i})$ where Ω is the set of all classes, *w_i_* is the weight or confidence associated with frame *i*, and **1**_*c*_(·) is the indicator function for class *c*. Output averaging requires an algorithm that returns a probability for each output class such as a NN. In output averaging, the probability values from the neural network were summed across the block and the label was selected to be the class with the greatest value, $c_{block}=\arg\;\max_{c\epsilon \Omega }\sum_{i=1}^{6}O_{c,i}$ where *O_c, i_* is the probability output of the neural network for class *c* at frame *i*.

### Mapping

We have extended NN sensitivity analysis to determine the spatial distribution of voxels that contribute to the classification of each class. The key idea is calculate the sensitivity (or derivative) of the neural network output (classes) with respect to each input (voxels). Let ***o*** be the vector of outputs and ***x*** be the vector of inputs. Then the sensitivity of output *k* to input *i* is defined by $S_{ki}=\frac{\delta o_{k}}{\delta x_{i}}$, which is the partial derivative of the output with respect to the input. Let ***w*** be the weight matrix from the hidden layer to the output layer and *w_kj_* be a single element of ***w*** corresponding to the weight on the network edge connecting output *k* with hidden node *j*. Similarly, let ***v*** be the weight matrix from the input layer to the hidden layer and *v_ji_* be a single edge weight. Then the partial derivative can be expressed as $\frac{\delta o_{k}}{\delta x_{i}}={o^{\prime }}_{k}\sum_{j=1}^{J}w_{kj}{y^{\prime }}_{j}v_{ji}$, where *J* is the total number of hidden units in that layer of the neural network, *o*′_*k*_ is the value of the derivative of the activation function at output *k*, and *y*′_*j*_ is the value of the derivative of the activation function at hidden neuron *j*. Finally, the entire sensitivity matrix can be expressed in matrix notation as ***S*** = ***O***′ × ***W*** × ***Y***′ × ***V***, where ***O***′ = *diag*(*o*′_1_, *o*′_2_, …, *o*′_*K*_) and ***Y***′ = *diag*(*y*′_1_, *y*′_2_, …, *y*′_*K*_).

Since the activation functions are generally non-linear, the sensitivity matrix becomes a function ***S***(***x***), where ***x*** is an input vector. However, the sensitivity matrix for a particular input vector can vary due to the stochastic nature of training NN. To compensate for this added variance, we trained 100 different nets and calculated the average sensitivity matrix ***S***_*avg*_ (***x***) across these samples.

We now have a sensitivity score for each voxel at all time points and for all output classes. However, we would like a measure of sensitivity only on voxels. Therefore, we calculated ***S*** for each point in the time series, and then computed the RMS average sensitivity matrix across all input vectors as $S_{RMS}=\sqrt{\sum_{n=1}^{N}S_{avg}{\left(x\right)}^{2}/N}$, where *N* is the number of input vectors (time points). ***S***_*RMS*_ gives a sensitivity value for each voxel with respect to all outputs. We then calculated the maximum sensitivity of each voxel across all outputs, i.e., ϕ_*i*_ = max_*k*=1…*K*_ *S*_*ki, RMS*_. This sensitivity was projected back into the volume anatomy to create a map of the relative incremental importance of each voxel's response to the classification decision.

In order to empirically determine a sensitivity threshold to eliminate irrelevant voxels, we propose an approach based on recursive feature elimination (RFE; Guyon et al., 2002) adapted to the feed-forward neural network. A similar approach was used by Formisano et al. (2008b) in conjunction with the weight vector of a regularized SVM. In RFE, a machine-learning algorithm is first trained on a full data set. Next, some ranking criterion is calculated for each input dimension. The dimension with the lowest rank is removed from the dataset (a fixed number or percentile of dimensions may be removed for speed reasons). Then, the machine-learning algorithm is retrained on the reduced dataset. This process can be repeated until all features have been removed. The performance of each subset can be calculated using a held-out test set to determine a good threshold to remove irrelevant voxels. We used the feed-forward neural network as our machine-learning algorithm, and the measure ϕ_*i*_ for our ranking criterion. For computational speed reasons as well as for inter-subject comparison, we used a fixed sensitivity threshold at each iteration to determine which features would be removed. This allowed us to bootstrap classifier performance on a held-out test set across all sessions to obtain 68% confidence intervals (Efron, 1979).

For a qualitative comparison, we created surface maps for the NN sensitivity analysis, GLM, and searchlight. These techniques cannot be used for a direct quantitative comparison because they present fundamentally different information. Similarly, the thresholds used for each map are not directly comparable. However, the thresholds have been selected based on standard practices for determining meaningful localization of function and information. For sensitivity analysis, recursive feature elimination was performed on each subject's volume sensitivity map until the bootstrapped classier performance fell significantly below (*p* = 0.05) the peak classifier performance. The resulting maps were projected onto their cortical surfaces and blurred along the surface using a 5 mm full-width half-maximum (FWHM) Gaussian kernel (voxel size is 2.5 mm). For GLM, a linear activation model was constructed using an explanatory variable for each character count. Processing of fMRI data was carried out using FEAT (FMRI Expert Analysis Tool) Version 5.98, part of FSL. Z (Gaussianized T/F) statistic images were thresholded using clusters determined by *Z* > 2.3 and a (corrected) cluster significant threshold of *P* = 0.05 (Worsley, 2001). For searchlight, we employed a 3 × 3 × 3 kernel and a linear SVM classifier using the PyMVPA toolkit (Hanke et al., 2009). The searchlight maps were thresholded at twice chance decoding accuracy (33%). These maps were then projected onto the Freesurfer generated surfaces for each subject. We attempted to use non-linear warping to create a group average, but we were not satisfied with the registration accuracy. In particular, there was a tendency to confuse activity on superior temporal areas with that on dorsal parietal regions. To average across subjects, therefore, we aggregated the maps across 10 anatomical labels automatically generated by Freesurfer during surface construction. To account for variations in the total surface area covered by the different maps, we calculated *percent coverage*, the fractional area of the thresholded map contained within each surface label, and bootstrapping was used to calculate 68% confidence intervals for all three approaches and all 10 surface labels.

## Results

### Classification accuracy

We built classifiers for three separate cases: with/without characters, 1–6 characters, and soldiers vs. insurgents. Recall that the with/without characters case has a block structure of 15 s. for each condition, and that both conditions contained images of the town. For this case, classification performance was excellent, with typical scores of 94–97% for the NN. This high performance was not too surprising, as there were strong low-level visual image differences between these two conditions. In contrast, the third case of distinguishing between soldiers and insurgents did not produce classification performance well above chance. Consequently, we focused our analysis on the second case, character counting where we did not distinguish between soldiers and insurgents.

**Figure 2:**
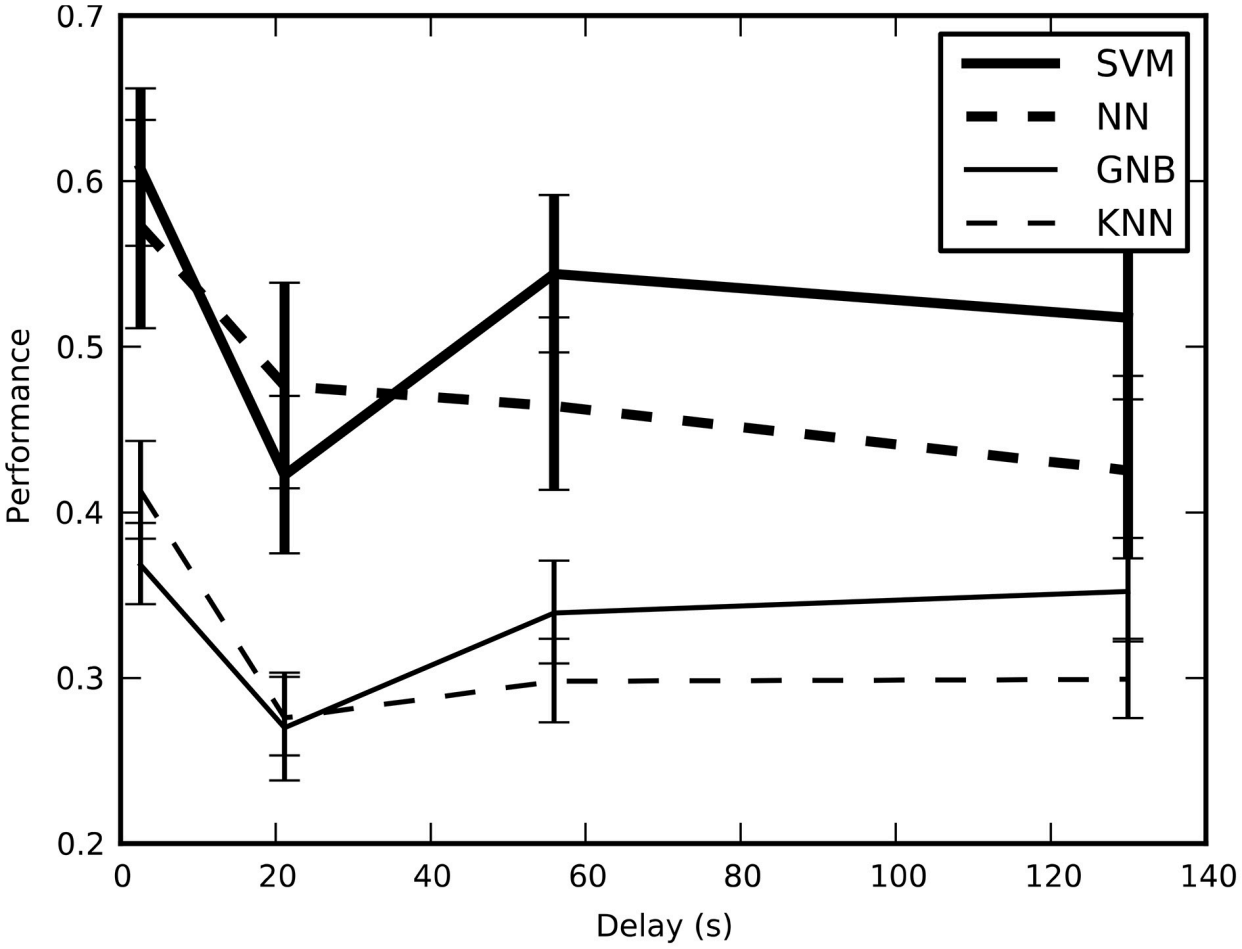
**The estimated performance of the classifiers averaged across all sessions and plotted across the four training-and-test-split methods; error bars show bootstrapped 68% confidence intervals**. There is a statistically significant drop in the estimated performance when the average minimum temporal delay increases from 2.6 to 21 s, though performance stays above the chance performance of 16.7%. This result confirms that short delays result in optimistic performance estimates because of temporal correlations.

We tested the cross-validated performance of the classifiers on four different training-and-test split methods to determine the method that would yield unbiased performance estimates with the lowest variance (Figure 2). Our results indicate that *block split* was the best method for estimating performance and subsequent results used this procedure.

To ensure that total contrast was not a confounding element in our results, we built a GLM with the total frame contrast as the target and the number of characters as explanatory variables. The resulting *p* values for this model are presented in the following table.

**Table:** 

**Character Count**	**1**	**2**	**3**	**4**	**5**	**6**
*p*-value	0.027	0.717	0.002	0.156	0.166	0.884

We found that character counts 1 and 3 did have a statistically significant correlation with total contrast. We also calculated the Pearson correlation coefficient between total contrast and character count while leaving out the 0 character blocks (*r* = 0.1598) and its significance (*p* = 0.1800), which did not show significant correlation. However, statistically significant trends do not necessarily drive high classifier performance, though they can contribute. To determine how much this affect could have contributed to classifier performance, we trained an SVM on only the total contrast information and measured its performance with cross-validation. We found the performance on only total contrast to be 25% and approximately 66% of the correct guesses were for character count 1 and 3. Therefore, the total contrast likely did impact classifier performance, but only for character counts 1 and 3. Furthermore, this cross-validated performance is significantly lower than the performance achieved by our machine learning algorithms on the fMRI data.

**Figure 3:**
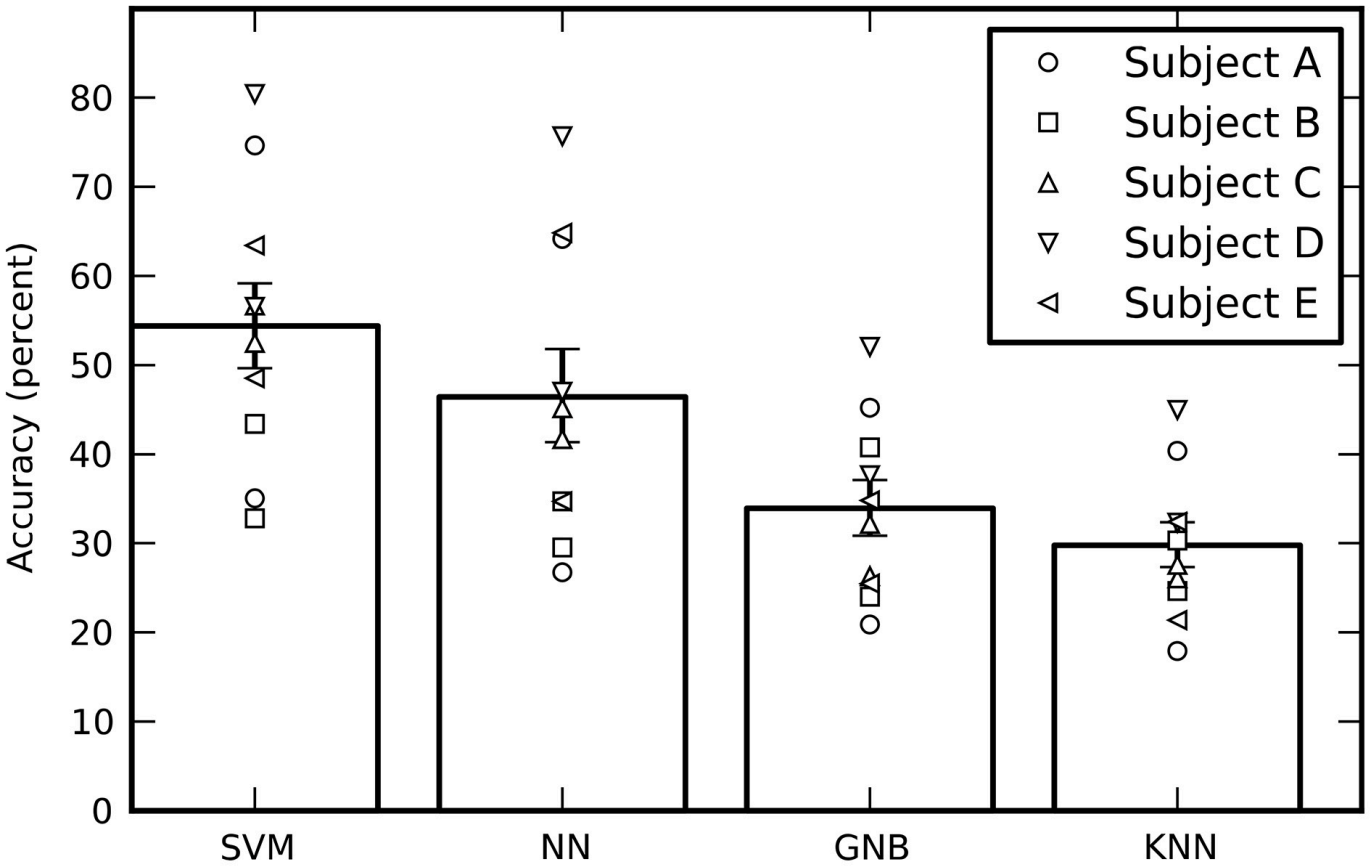
**The estimated performance of all four classifiers averaged across all sessions**. The performance of individual sessions are indicated by the symbols. Each subject performed two sessions and there are therefore two symbols per subject. The performances estimates were bootstrapped across sessions in order to obtain 68% confidence intervals. While the SVM had the best average performance, all four classifiers performed well above a chance performance of 16.7%.

Averaged across all 10 sessions (five subjects with two sessions each), the cross-validated performance estimates of all four classifiers are significantly above chance, where chance is one out of six = 16.7% (Figure 3). The SVM had the best performance, followed by the feed-forward NN (without using our new output processing techniques; see below). The performance of all four independent classifiers being above chance increases confidence in the results, however the GNB and KNN classifiers will not be discussed further as their performance was significantly below the SVM and NN. There is considerable variation in performance between sessions for the same subject, as well as variation in average performance between subjects.

It is also worth noting the computation time of these algorithms in practice. The average training time was 0.683 ms per example for the SVM, 121.299 ms per example for the NN, 0.073 ms per example for the GNB, and 0.044 ms per example for the KNN. The training time of the NN is this ~2 orders of magnitude slower than the SVM. Nevertheless, the full NN cross validation procedure still only took approximately 10 min per session. The average decoding time was 0.431 ms per example for the SVM, 0.197 ms per example for the NN, 0.172 ms per example for the GNB, and 0.466 ms per example for the KNN. Unlike training times, the NN is the second fastest at decoding.

**Figure 4:**
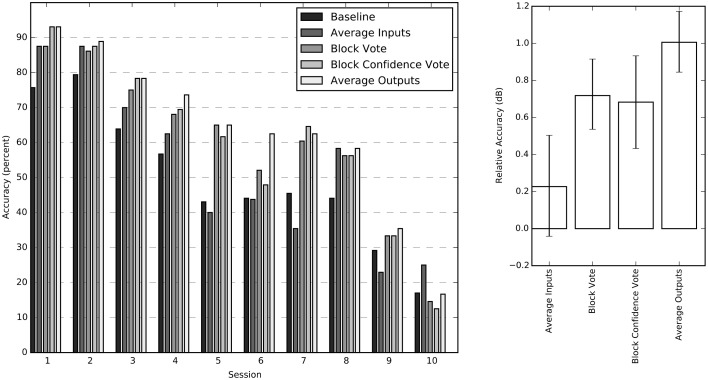
**Individual session accuracies and relative accuracy for all four averaging methods**. The sessions have been sorted by average performance for improved readability. The chance probability for all sessions is 16.7%. The chart on the right shows the impact of the individual aggregation methods calculated relative to the baseline score as 10 log (score/baseline) for each session. These relative accuracy scores (in dB) are averaged across all sessions and bootstrapped to obtain 68% confidence intervals.

We tested four different methods for exploiting the block structure of the stimulus to improve classification accuracy: input averaging, block vote, confidence vote, and output averaging. Since confidence vote and output averaging require an estimate for the probability of each output label, only the feed-forward NN was considered for this comparison. Both block voting methods, and output averaging improved session performance significantly over simple input averaging. The output averaging method had the greatest average improvement (Figure 4).

**Figure 5:**
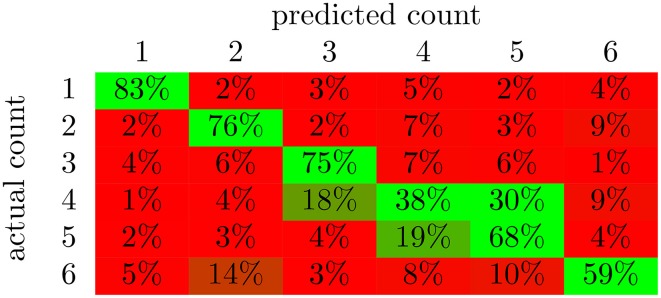
**The average confusion matrices for the feed-forward NN with output averaging across all subjects**. The value in cell (i,j) of the matrix is the percent of examples from class i that were labeled as class j; values along the diagonal indicate correctly classified examples while the rest indicate incorrectly classified examples. The color of the cell indicates deviation from chance probability (16.7%); greener cells indicating values above chance, and redder cells indicating values below chance.

From the confusion matrix in Figure 5, we see that the classifier is best at detecting the presence of a single character. In fact, there are relatively few cases of confusion between one and two characters. Apparently, these two situations evoke very different responses in the brain. Also, note that the majority of the incorrect responses lay just off the main diagonal. These responses correspond to the classifier being wrong by a single character in its classification. 1 and three characters were classified with the highest accuracy. This is likely due in part to the correlation with total scene contrast. However, note that two characters were also classified with high accuracy and yet had the second lowest *p*-value for contrast correlation.

**Figure 6:**
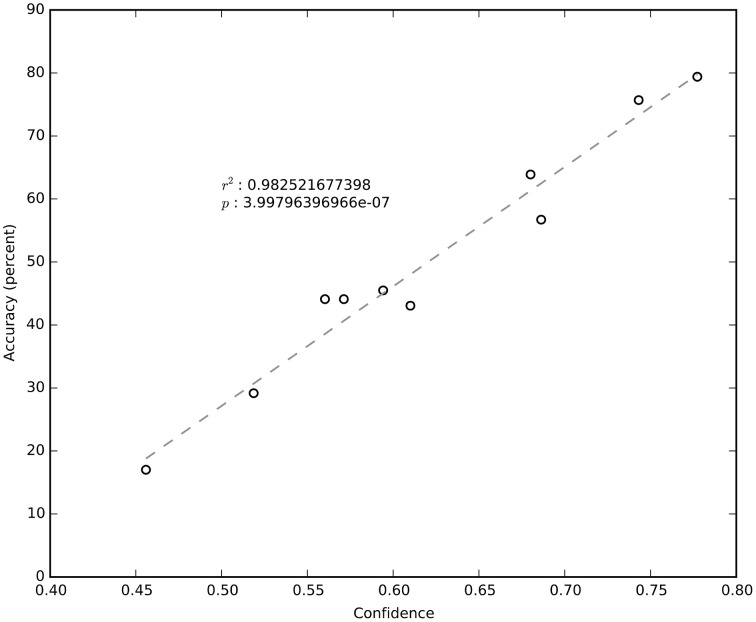
**Cross-validated session accuracy plotted against average session confidence**.

It is clear from Figure 4 that not all sessions performed equally well. Even for the same subject, session performance varied significantly. We found that the average confidence (i.e., the probability of the chosen label) returned by the NN was very significantly correlated (*R*^2^ = 0.98; negligible *p*) with the network's cross-validated performance (Figure 6). The confidence measure was calculated without knowledge of the labels and thus provides a measure of the quality of the data being classified as well as an estimate for how well the NN has estimated the joint probability distribution.

### Mapping

**Figure 7:**
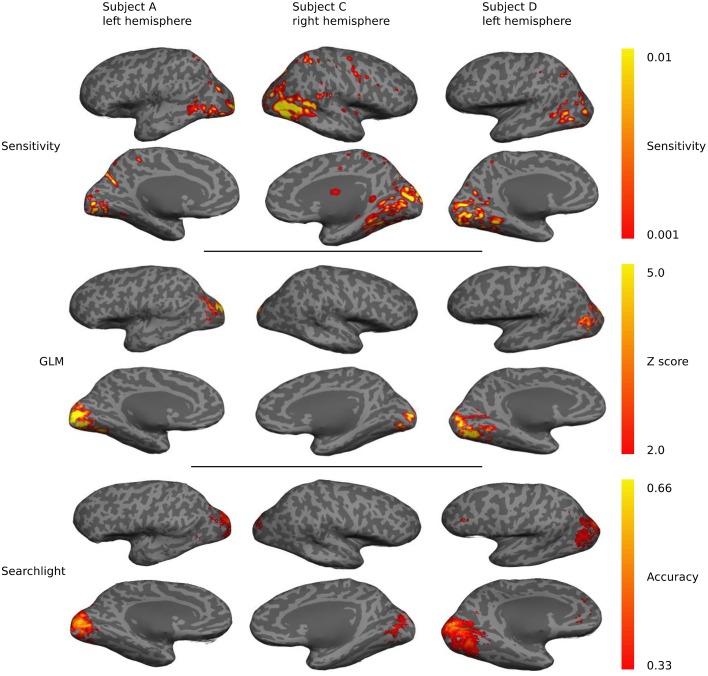
**A qualitative comparison of sensitivity, GLM linear-response Z-statistic, and searchlight accuracy maps projected onto semi-inflated cortical surfaces for three different subjects**. The maps are roughly similar across subjects and hemispheres, but substantial individual variations are evident.

Sensitivity maps for individual subjects show a preponderance of classification sensitivity in lateral occipital areas, ventral early visual areas, and dorsal parietal lobe (Figure 7). Subjects also displayed small regions of high sensitivity in portions of temporal and frontal cortex. There is significant overlap between the sensitivity, GLM, and searchlight maps, but the sensitivity maps show greater contributions from anterior brain regions.

**Figure 8:**
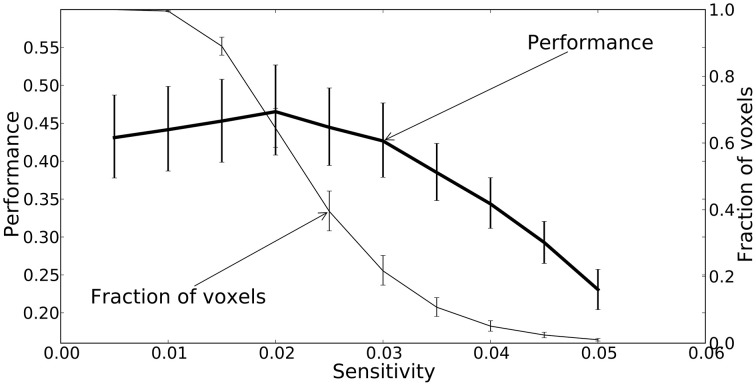
**A plot of the feedforward neural network estimated performance and the fraction of voxels remaining at each iteration of the recursive feature elimination procedure**. The fraction of voxels is calculated with respect to the 2000 voxels selected by ANOVA. The performance estimates and voxel counts were bootstrapped across sessions in order to obtain 68% confidence intervals.

All three mapping techniques are intended to help localize function or information, however the meaning of the values in these maps are not equivalent, and neither are their thresholds. The thresholds were chosen based on accepted practice for their associated technique, but they are not statistically equivalent and should only be used for qualitative comparison. Sensitivity threshold values were determined using a recursive feature elimination approach (Section Mapping). GLM linear-response Z-statistic maps and searchlight accuracy maps are also presented for comparison. The sensitivity maps were thresholded using the recursive feature elimination technique described in the Methods. The Z statistic images were thresholded using clusters determined by *Z* > 2.3 and a (corrected) cluster significant threshold of *P* = 0.05 (Worsley, 2001). The searchlight maps are thresholded at twice chance probability (33%). Figure 8 presents the performance of the NN and the fraction of voxels remaining after each iteration. Greater than half the voxels can be removed without significant loss of classification performance.

**Figure 9:**
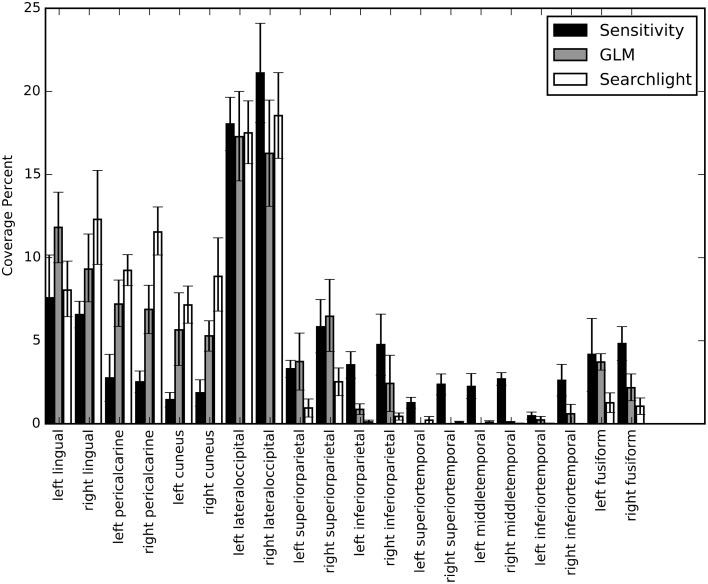
**A bar graph depicting the coverage percent from the sensitivity, GLM, and searchlight maps across automatically generated labels from Freesurfer**. The coverage percent is the percent of the map contained within that area. In this way, the variation in total map size between approaches is controlled for and specificity and coverage of the maps can be directly compared. Coverage percentages were bootstrapped across sessions to provide confidence intervals.

We calculated percent coverage for each of the three mapping methods on Freesurfer anatomical surface labels (Section Mapping). Results were averaged across all sessions and bootstrapped to obtain 68% confidence intervals (Figure 9). These numbers agree with our observations on the individual surface maps; there is substantial overlap between all three maps in lateral occipital and lingual cortex. Elsewhere the mapping methods show different patterns of response. For example, early visual cortex, roughly demarcated by the pericalcarine and cuneus labels, shows greatest classification sensitivity by the searchlight technique, intermediate response based on GLM, and relatively low information content based on our NN sensitivity metric. Interestingly, several temporal lobe regions show greater sensitivity based on the NN metric than either of the others. To determine if the sensitivity in these regions is meaningful, we estimated the performance of the NN on a subset of the original voxels constructed by taking all of the voxels considered significant by the sensitivity analysis and removing all those voxels considered significant by GLM. The cross-validated performance on this subset averaged across all sessions was 25% (with *p* < 0.05 for all sessions). While the performance dropped substantially, these voxels were still able to classify character count significantly above chance.

## Discussion

We set out with the practical goal of decoding the subject's cognitive state associated with viewing a number of characters from time series of functional images. Using a combination of standard and novel machine-learning methods, we were able to extract this information with accuracy that varied from well above chance to nearly perfect (Figure 4), depending upon session and machine-learning algorithm. For the neural network results, we then presented a novel approach to relating the network's decision-making sensitivity back to brain anatomy of the individual. These sensitivity maps suggest that a more widespread and diverse network of brain regions encoded the cognitive state, which is consistent with the complex nature of the VE stimulus.

The work described in this paper expands the opportunities for utilizing VEs for scientific inquiry in cognitive neuroscience. The design of the stimulus provided a balance between realism and experimental control so that quantitative analysis of the fMRI data stream achieved a degree of confidence ranging from satisfactory (well above chance) to very high. Care was taken to preserve as much of a natural experience as possible. For example, we never exposed the subject to disruptions in the experience of being present in a virtual environment, yet the stimulus had an otherwise classic block design. And the synthesized video stream never showed static images at any time, which rarely occur under natural conditions. We also eschewed averaging data between different subjects in accordance with one of our goals: modeling individuals for therapies and learning regimens, including utilizing real-time fMRI.

Despite these seemingly greater challenges, we were able to achieve classifier performance that was significantly above chance with all four of the MVPA methods we tested. More importantly, for the two strongest methods, support-vector machines and artificial NN, the classifier performance was sometimes good enough to enable practical applications. This is especially impressive given that the cognitive states being discriminated were not based on differing object categories (e.g., houses, faces, tools, etc…) that often activate brain regions with limited anatomical overlap (Hanson et al., 2004), but rather were from a single object category, viz. combatants, and differed only in number of combatants.

We also discovered that the performance of classic feed-forward NN, which have been somewhat neglected lately in favor of SVM, can be competitive with SVM on the data in this study. While the inherent properties of SVM make it well suited to sparse representations (small number of object categories vs. large number of voxels), NN provide a more general method that can (in principle) capture more subtle features given enough data. Moreover, NNs provide probability values that can be used to further improve classification performance. Looking to the future, building NNs using “deep learning” (Hinton et al., 2006) has been shown repeatedly to outperform SVM on many types of data (Cirşan et al., 2012). Even greater classifier accuracy may be possible with such methods applied to VE data.

Classifier performance will be important for both on-line use of fMRI in brain-computer interfaces (BCI), such as PTSD therapy, as well as for off-line creation of brain maps using sensitivity analysis. The techniques block voting, confidence voting, and output averaging (see Section Block Integration), all improved performance over the baseline classifier performance as well as over input averaging. The concept of using the output of the classifier to ascribe confidence (see Section Classifier Probability and Confidence) to each output could be very useful for differentiating the reliability of entire sessions. Similarly, any confidence measure could be quite valuable in BCI applications in which low confidence frames could be weighted by confidence to reduce their influence and/or dropped entirely from any on-line decision-making by the BCI software.

Classification sensitivity in early retinotopic visual areas and lateral-occipital areas suggests that retinotopic organization is important to decoding group size for our VE stimulus. Because LO combines object-selectivity with retinotopic specificity (Sayres and Grill-Spector, 2008), different group sizes could evoke complex but stereotypical patterns of responses in LO (and other retinotopically organized areas) as subjects visually interrogate the stimuli with a sequence of eye movements. Regions in the parietal cortex have been shown to be involved in mental arithmetic and magnitude judgment (Rickard et al., 2000) which may also play some role in decoding group size. More recent research suggests this region may even contain a topographic representation of numerosity (Harvey et al., 2013). There is some debate as to whether this topographic map represents numerosity or sensory processing (Gebuis et al., 2014), but it would be useful for decoding group size regardless.

Integrating information from the whole brain improves decoding accuracy, but it makes interpreting functional localization problematic. From our sensitivity analysis, we see that regions associated with low-level vision, higher-level object-recognition, and potentially even cognitive representations of numerosity all contributed to decoding. However, the sensitivity analysis does not necessarily tell us how these regions contributed. Eye movements and other behavioral responses as the subjects visually interrogate the stimuli could induce reliable and complex patterns of activation in all of these areas. For example, our control analysis indicates that low-level contrast features may have partially, but not entirely contributed to decoding. Similarly, increased eye movements could create a higher variance of activation in retinotopic visual areas. If this behavior is reliable and consistent, the machine learning algorithms will learn to use that information to help decode the state. At this early stage, we did not collect eye tracking data during our experiments to evaluate to what extent this contributed to decoding. Eye-movement information is not obviously correlated with character count, but rather the cognitive evoked in the subject by the VE: being in a town and freely viewing a specific number of characters. It is this VE-specific state that we are interested in decoding. Such goal-driven decoding should be more useful for training and therapy exercises where the underlying neural mechanisms may not yet be well understood. However, neuroscientific studies looking to leverage VEs and sensitivity mapping for functional localization must still be careful to balance realism with control to avoid these kinds of confounds when interpreting their results.

The GLM produced Z-statistic maps indicate significant activation only in early ventral visual areas and lateral occipital regions. Searchlight produced results qualitatively similar to GLM, suggesting that the expansion from a single voxel with GLM to a 3×3×3 set of voxels in searchlight was not sufficient to capture potentially important long-range multi-voxel response patterns identified by the NN sensitivity analysis. Therefore, we conclude that extracting response patterns by performing classification on voxels selected from a spatially diverse collection of voxels captures potentially important brain information missed by both GLM and searchlight (Figure 9).

Note that the information contained in the maps is quite different, making them difficult to compare directly. The Z-statistic maps tell us how well individual voxels agree with a hypothetical model, the searchlight maps tells us how well small localized groups of voxels are able to decode the desired brain state, and the sensitivity maps tells us how much individual voxels contribute to a spatially-distributed decoding decision. We do not have a practical way to calculate *p*-values for individual voxels with the sensitivity analysis so care must be taken when interpreting the results. However, a qualitative comparison of the techniques is still useful. While we are unable to calculate the significance of individual voxels for our sensitivity analysis, the comparison shows that the resulting sensitivity maps highlight regions consistent with accepted mapping techniques where per voxel significance calculations are possible. This increases our confidence that the areas indicated by the sensitivity analysis, but not the other techniques, likely do contain information relevant for decoding the subject's brain state and could merit further investigation.

In conclusion, it is possible to extract useful information from fMRI data obtained using a realistic virtual environment stimulus using machine-learning methods. NN, supplemented by some averaging techniques, performed particularly well. The resulting classification data, moreover, can be mapped onto the brain using a novel form of sensitivity analysis. These methods open up new possibilities for the use of VEs in both neuroscience research and in clinical applications.

### Conflict of interest statement

The authors declare that the research was conducted in the absence of any commercial or financial relationships that could be construed as a potential conflict of interest.
