# Supplementary material for: Accurately decoding visual information from fMRI data obtained in a realistic virtual environment
Source: Front Hum Neurosci. 2015 Jun 9;9:327. doi: 10.3389/fnhum.2015.00327 (PMC4460535; doi:10.3389/fnhum.2015.00327)
Supplement: Supplementary file 1 [file AppendixA.PDF]

1 Appendix A

$BlockVote(Y, L)$

Input: Y: the list of decoded labels for each frame in the block
L: the set of all labels

Output: the aggregate label for the block

for each label $l \in L$ **do**
$C_l = 0$
end for
for each frame i **in the block**:
$l = Y_i$
$C_l = C_l + 1$
end for
return $argmax(C)$

$ConfidenceVote(Y, L, W)$

Input: Y: the list of decoded labels for each frame in the block
L: the set of all labels
W: the set of confidence scores for each label and each frame in the block

Output: the aggregate label for the block

for each label $l \in L$:
$C_l = 0$
end for
for each frame i **in the block**:
$l = Y_i$
$C_l = C_l + W_{i,l}$
end for
return $argmax(C)$

$OutputAverage(Y, L, W)$

Input: Y: the list of decoded labels for each frame in the block
L: the set of all labels
W: the set of confidence scores for each label and each frame in the block

Output: the aggregate label for the block

for each label $l \in L$:
$C_l = 0$
end for
for each frame i **in the block**:
for each label $l \in L$:
$C_l = C_l + W_{i,l}$
end for
end for
return $argmax(C)$

$NetworkJacobian(W, V, x)$

Input: W: weights from hidden layer to output layer
V: weights from input layer to hidden layer

x: input vector

Output: Jacobian matrix for the network evaluated at the given input vector

for $i = 1$ **to** $length(x)$:
    for $k = 1$ **to** $rows(W)$:
        $o = 0$
        $d = 0$
        for $j = 1$ **to** $cols(V)$:
            $o = o + W_{k,j} \cdot hidden\_transfer(V_{j,i} \cdot x_i)$
            $d = d + W_{k,j} \cdot V_{j,i} \cdot hidden\_transfer\_deriv(x_i)$
        $J_{i,k} = output\_transfer\_deriv(o) \cdot d$
        end for
    end for
end for
return J

$NetworkTotalSensitivity(X, Y)$

Input: X: list of all input examples
Y: list of correct labels for each input example

Output: Matrix representing total network sensitivity for each output and input
Network accuracy

for $i = 1$ **to** 100:
    $W_i, V_i, a_i = train\_network(X, Y)$
end for
for $i = 1$ **to** $length(x: x \in X)$:
    for $k = 1$ **to** $rows(W_1)$:
        $ss_{i,k} = 0$
    end for
end for
for each example $x \in X$:
    for $i = 1$ **to** $length(x)$
        for $k = 1$ **to** $rows(W_1)$:
            $s_{i,k} = 0$
        end for
    end for
    for $i = 1$ **to** 100:
        $s = s + NetworkJacobian(W_i, V_i, x)$
    $m = \frac{s}{100}$
    $ss = ss + m * m$
end for
return $sqrt\left(\frac{ss}{|X|}\right), \frac{a_i}{100}$

$NetworkSensitivity(X, Y)$

Input: X: list of all input examples
Y: list of correct labels for each input example

Output: Vector representing maximum network sensitivity for each input
Network accuracy

$T, a = NetworkTotalSensitivity(X, Y)$

for $i = 1$ **to** $rows(T)$:

$max = 0$

for $j = 1$ **to** $cols(T)$:

if $T_{i,j} > max$:

$max = T_{i,j}$

end if

end for

$S_i = max$

end for

return S, a

$RecursiveFeatureElimination(X, Y, t, s)$

Input: X: list of all input examples

Y: list of correct labels for each input example

t: decoding accuracy threshold

s: step size for reducing input dimensions

Output: Vector representing network sensitivity for each input after recursive feature elimination

$i = 0$

$X_i = X$

do

$i = i + 1$

$S_i, a_i = NetworkSensitivity(X_{i-1}, Y)$

$d = ArgSort(S_i)$

$k = \{d_j : j = 1 \text{ to } |d| - s\}$

$X_i = \left\{\{x_j : j = 1\ to\ |x| \text{ and } j \in k\} : x \in X_{i-1}\right\}$

while $a_i > t$

return S_{i-1}
